# Anti-proliferative and pro-apoptotic effects induced by simultaneous inactivation of HER1 and HER2 through endogenous polyclonal antibodies

**DOI:** 10.18632/oncotarget.19958

**Published:** 2017-08-03

**Authors:** Narjara González Suárez, Gretchen Bergado Báez, Mabel Cruz Rodríguez, Amelia Gutiérrez Pérez, Lisset Chao García, Diana Rosa Hernández Fernández, Judith Raymond Pous, Belinda Sánchez Ramírez

**Affiliations:** ^1^ Tumor Immunology Direction, Molecular Immunology Institute, Center of Molecular Immunology, Havana 11600, Cuba; ^2^ System Biology Direction, Molecular Immunology Institute, Center of Molecular Immunology, Havana 11600, Cuba

**Keywords:** HER1, HER2, polyclonal antibodies, receptor degradation, apoptosis

## Abstract

The human epidermal growth factor receptor (HER1) and its partner HER2 are extensively described oncogenes and validated targets for cancer therapy. However, the effectiveness of monospecific therapies targeting these receptors is hampered by resistance emergence, which is frequently associated with the upregulation of other members of HER family. Combined therapies using monoclonal antibodies or tyrosine kinase inhibitors have been suggested as a promising strategy to circumvent this resistance mechanism. We propose an alternative approach based on simultaneous inactivation of HER1 and HER2 by multi-epitope blockade with specific polyclonal antibodies induced by vaccination. Elicited antibodies impaired both receptors activation and induced their degradation, which caused the inhibition of down-signaling cascades. This effect was translated into cell cycle arrest and apoptosis induction of human tumor cells. Elicited antibodies were able to reduce the viability of a panel of human tumor lines with differential expression levels of HER1 and HER2. The most significant effects were obtained in the tumor lines with lower expression levels of both receptors. These new insights would contribute to the rational design of HER receptors targeting multivalent vaccines, as an encouraging approach for the treatment of cancer patients.

## INTRODUCTION

The human epidermal growth factor receptor (EGFR or HER1) family comprises four receptors (HER1-4) with tyrosine kinase activity [1]. HER1 and HER2 deregulation, resulting from overexpression or mutation of these receptors has been implicated in several types of malignancies [2] and is often associated with poor prognosis of the disease. Hyperactivation of downstream signaling pathways such as mitogen activated protein kinases (MAPK), phosphoinositol kinase 3 (PI3K) and signal transducer and activator of transcription 3 (STAT3), promotes tumor cells growth, differentiation, migration, and evasion of apoptosis [3]. Some reports have also described the association between HER1 and HER2 expression and activation with a decreased expression of MHC-I [4, 5]. Additionally, it has been described the linkage between HER1 activation and the increased expression of immune-suppressive molecules like PD-L1 [6], as well as the secretion of inflammatory cytokines in tumor cells [7, 8]. Then, these oncogenes not only play a key role in tumor initiation and progression, but also contribute to evade the control exerted by the immune system. The connection of HER1 and HER2 with the mentioned hallmarks of cancer, explains why tumors often create addiction to these oncogenes, as well as the number of targeting therapies that have been designed to prevent their function [9]. Taking into account that both tumor biology and immune system status can influence how patients respond to anticancer drugs, therapies that prevent HER1 or HER2 activation can be seen as modulators of both processes. Some monoclonal antibodies (MAbs) and tyrosine kinase inhibitors (TKIs) have been already approved for cancer treatment [3, 10, 11]. Nevertheless, despite recognized results achieved in clinic with cetuximab and trastuzumab (Herceptin), the most advanced MAbs targeting HER1 and HER2, respectively, only some patients are benefited. The efficacy of these MAbs has been associated with high levels of expression, and absence of mutations in the targeted receptor [12, 13]. Furthermore, in most cases resistant variants emerge, limiting the clinical success of these antibodies [14, 15]. Due to the redundancy in the signalling cascades recruited by HER receptors, one of the resistance mechanisms described is the up-regulation of other member of the family, which provides compensatory survival signals [12, 15]. Hence, blocking several epitopes in more than one HER family member could be a striking strategy to avoid resistance associated with the expression of these compensatory molecules.

Current preclinical data suggest that targeting HER1 or HER2 using different MAbs or a combination of MAbs with TKI are better alternatives than monospecific therapies. Maron et al. demonstrated that combination of two antibodies targeting HER1 and HER2 impairs receptors dimerization, enhances receptors degradation and synergizes in tumor growth inhibition in preclinical models [16]. This promising result has been already translated to the clinic, combining cetuximab and trastuzumab in a phase 1-2 clinical trial. Unfortunately, although some patients were stabilized, the treatment was stopped due to the frequency and severity of adverse events [17].

Considering previous preclinical and clinical data, we propose an alternative strategy based on simultaneous inactivation of HER1 and HER2 by specific polyclonal antibodies (PAbs) induced by vaccination. Protein-based vaccines use low dose of antigens to induce long term polyclonal immune response, while its low toxicity has been demonstrated in the clinic [18]. We found that neutralizing PAbs provoked the inhibition and degradation of both receptors, which caused cell cycle arrest and apoptosis induction in treated tumor cells. Also, we suggest that tumor biology disturbance by multi-epitope and multi-antigen targeting can circumvent the need of high expression levels of HER1 and HER2 to induce cytotoxicity. This opens the possibility to consider our approach for the development of a novel cancer vaccine.

## RESULTS

### Induction of endogenous anti-HER1/HER2 polyclonal antibodies

**Figure 1:**
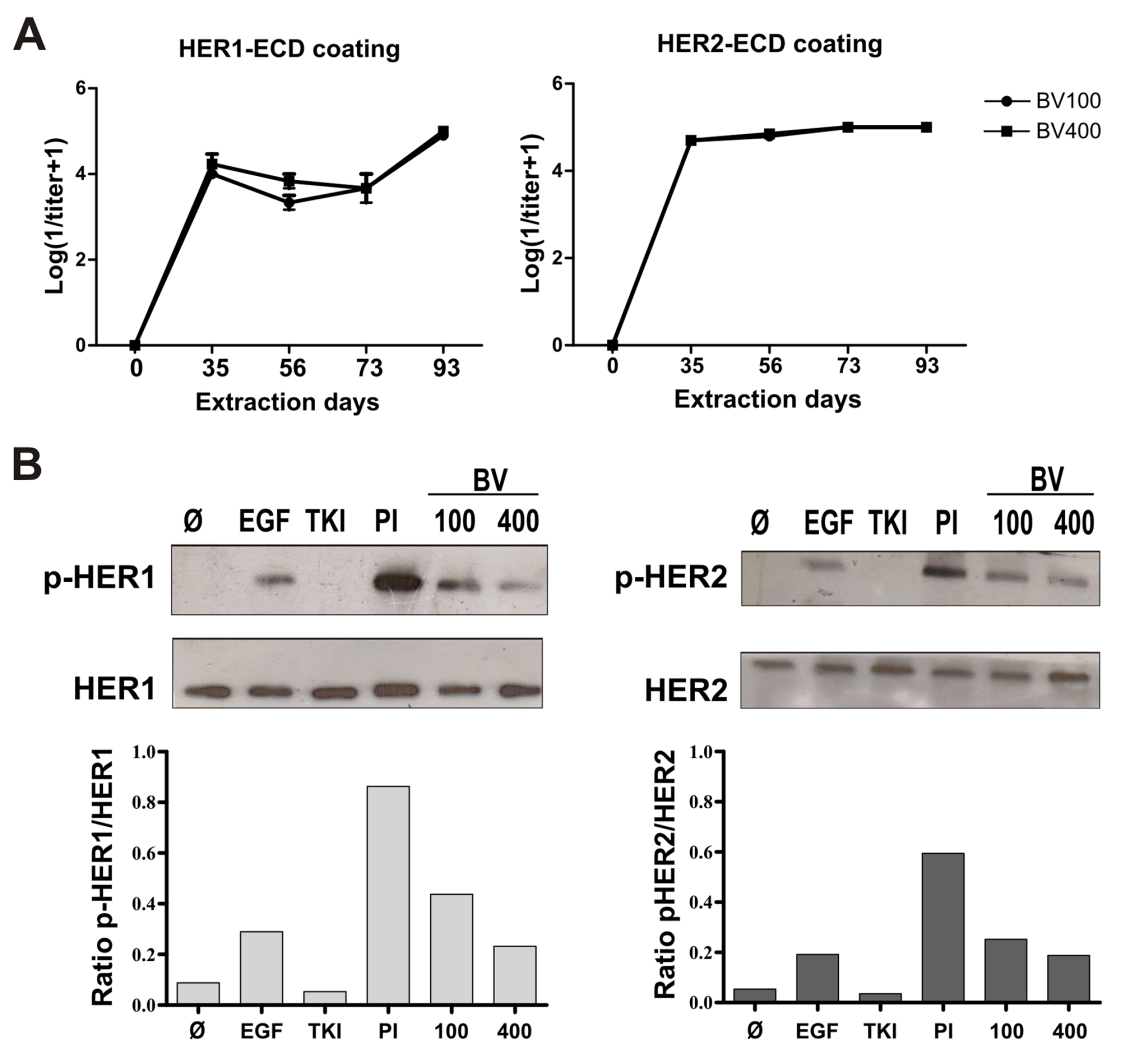
Induction of specific PAbs against HER1 and HER2 **(A)** Sera from BALB/c mice (n=5) immunized with bivalent vaccine including a mixture of 100μg (BV100) or 400μg (BV400) of HER1-ECD and HER2-ECD, were obtained on days -2, 35, 56, 73 and 93. Quantification of specific IgG antibodies against HER1 or HER2 was performed by ELISA. Data was Log transformed (1/titer + 1) for graphic representation. Non statistical differences among the groups were found using the non-parametric Mann-Whitney U test. **(B)** H292 tumor cells were incubated for 30 minutes with immune sera obtained from mice immunized with BV100 or BV400, at extraction day 56 (diluted 1:100). Cells were stimulated for 10 min with EGF (100ng/mL). HER1 and HER2 expression and phosphorylation state was analysed by Western blot. Pooled pre-immune sera (PI) was included as negative control, while Tyrosine kinase inhibitor (TKI) AG1478 (1μM) was used as positive control. For densitometry the program ImageJ was employed. Representative results of one of two performed experiments are shown.

To evaluate if simultaneous immunization with equivalent dose (100μg) of the ECDs of HER1 and HER2 affected the antibodies response against each molecule, bivalent and monovalent immunizations were compared. As a result, polyclonal antibodies were induced by immunization with the bivalent vaccine, which were specific against both receptors (Figure 1A). Also, bivalent and monovalent compositions were equally immunogenic in terms of antibodies induction (Supplementary Figure 1A). The specificity of the response was not related with the adjuvant, since anti-HER1/HER2 antibodies were not induced in mice immunized only with VSSP (Supplementary Figure 1B). Additionally, we tested whether an increase in the antigens dose could induce a better humoral response in terms of specific PAbs titers and quality. As shown in Figure 1A, there were no changes in antibodies titers when we increased the antigen doses four times. Next, we compared the quality of PAbs induced with the mixture of 100μg or 400μg of both ECDs attending to their capacity to impair ligand-induced activation of the targets. Noticeable, in these experiments we observed a higher phosphorylation of both HER1 and HER2 in the cells incubated whit pre-immune sera and pulsed with human EGF, than cells stimulated only with this ligand. This effect was related to murine EGF present in mice sera (both pre-immune and immune) [19]. According to this fact, pre-immune serum was considered as negative control in our experiments. As shown in Figure 1B, PAbs induced by both bivalent compositions decreased the phosphorylation of HER1 and HER2 within 30 minutes of incubation, compared with pre-immune sera. This inhibitory effect was dependent on the incubation time, since the phosphorylation of both receptors was completely abrogated after one hour of incubation (Supplementary Figure 1C). However, PAbs obtained with 400μg of both ECDs, prevented ligand-dependent activation of HER1 more effectively than the antibodies induced with 100μg of the antigens. In the case of HER2 the differences between both formulations were less pronounced (Figure 1B). Therefore we selected sera from mice immunized with 400μg of both ECDs to further characterize the impact of PAbs in HER1/2 signaling, and its implications for tumor cell viability.

**Figure 2:**
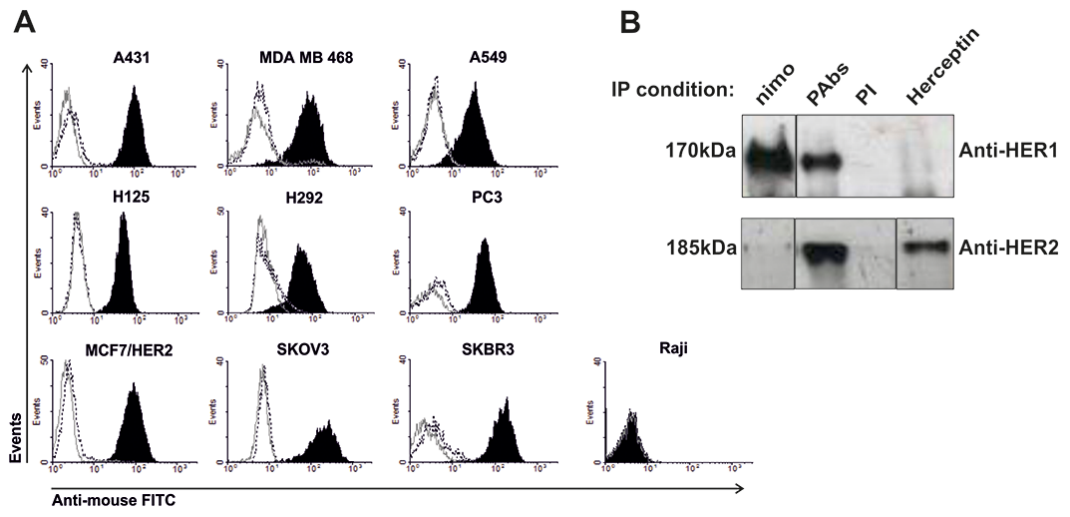
Recognition of tumor lines expressing HER1/HER2 **(A)** Cells lines were incubated with the pooled immune sera (black-filled histogram) collected from day 35 and diluted 1: 200 in PBS at 2% of SCF, followed by goat anti-mouse IgG FITC (gray solid line) stain. Pre-immune serum (black dotted line) was used as negative control for recognition. **(B)** A431 lysate was incubated overnight with immune sera to immunoprecipitate HER1 and HER2. Detection of these receptors was performed by immunoblotting. Pre-immune serum was used as negative control, while MAbs nimotuzumab (nimo) and Herceptin were used as positive controls for specific HER1 and HER2 (respectively) precipitation. The pictures were processed to alter the order of the immunoprecipitation reactions (IP), to get a match between both immunoblotting (IB) conditions.

As the immunizations were performed with truncated forms of these receptors, we confirmed the capacity of elicited PAbs to recognize a panel of tumor cell lines expressing both receptors. Ten human cell lines with different expression levels of HER1 and HER2 were tested. This differential expression was confirmed using the MAbs nimotuzumab and Herceptin, respectively (Supplementary Figure 2). All cell lines were recognized by induced PAbs regardless their receptors expression levels, apart from Raji (B cell lymphoma) which was included as a negative control (Figure 2A). The PAbs were also able to precipitate full receptors from A431 lysates which confirmed the specificity of the immune response (Figure 2B).

Since tumors can actively compromise the immune system of the host, we evaluate the capacity of the bivalent vaccine to induce specific PAbs in tumor-bearing mice. To this aim, mice were inoculated subcutaneously with F3II tumor cells and further immunized with the bivalent vaccine. Regarding the magnitude of the antibodies response, we found no differences between the titers reached in healthy or tumor-bearing mice for any of the receptors. Also, PAbs generated in both groups were able to recognize tumor cell lines expressing HER1 and HER2, in a similar way (Supplementary Figure 3).

### Induced Abs decrease HER1/HER2 expression and inhibit downstream cascades, impairing the viability of H292 tumor cells

**Figure 3:**
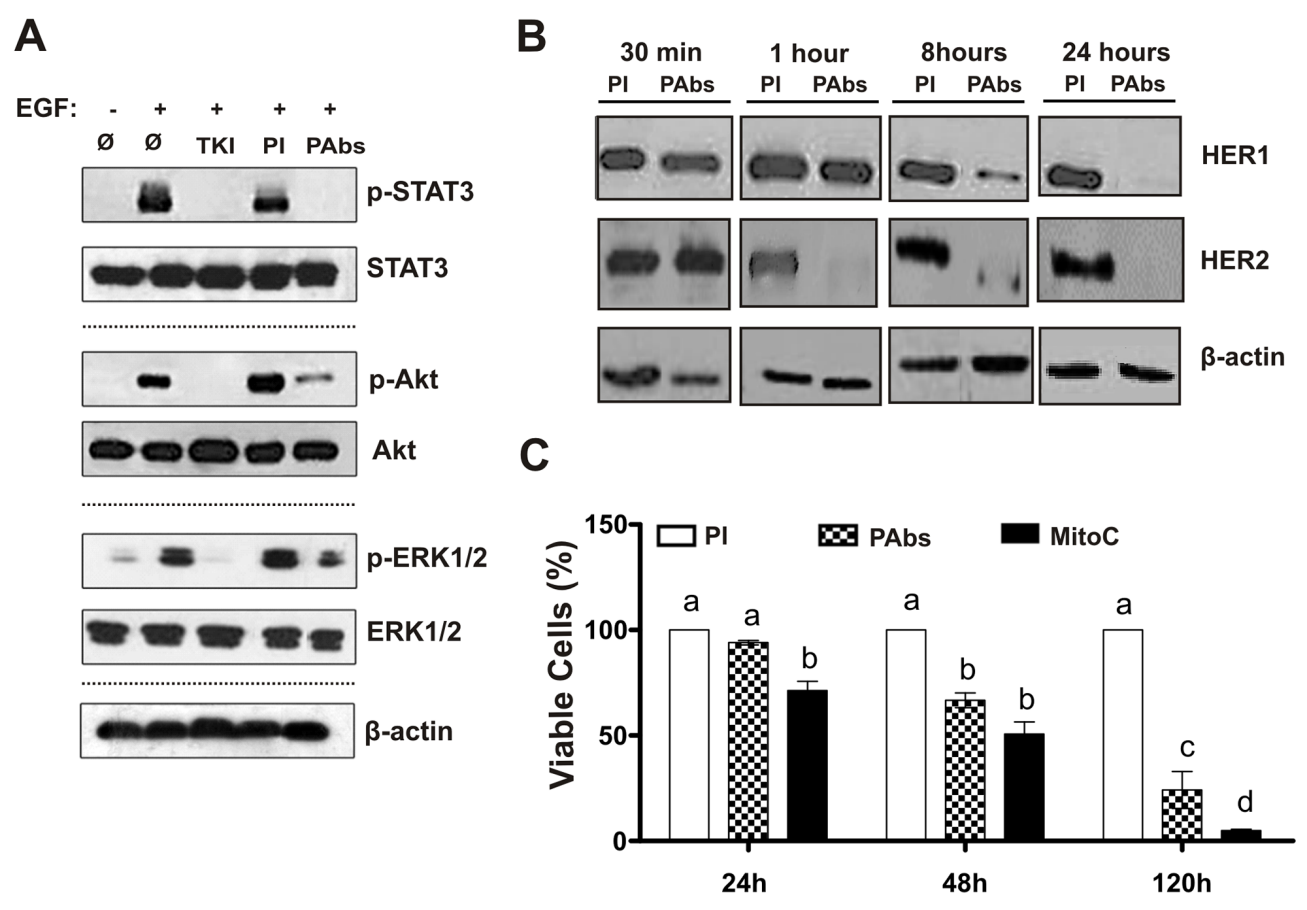
Induction of HER1/HER2 degradation, inhibition of downstream cascades signalling and reduction of H292 cells viability **(A)** H292 cells were incubated during 8 hours with pooled immune sera (PAbs) from extraction day 56 (diluted 1:100), and further stimulated with EGF (10 min, 100ng/mL). STAT3, Akt and ERK1/2 expression and phosphorylation state was analysed by Western blot, and compared with cells incubated with pre-immune sera (PI) used as negative control. AG1478 (TKI) (10μM) was included as a positive control of receptor inhibition. **(B)** HER1 and HER2 expression was assessed by Western blot at different end points (from 30 minutes to 24 hours) in H292 cells treated with PAbs from day 35, diluted 1: 100. Cells treated with PI were considered as negative control. **(C)** Cells viability was determined by MTT assay after 24, 48 and 120 hours of incubation with PAbs (pattern box) or PI (white box) diluted 1: 20 and heated at 56°C for 30 minutes to inactivate the complement. Mitomycin C (MitoC, 25μg/mL) was considered as positive control of cell viability reduction (black box). The 100% of viability was referred to PI incubation. Mean ± S.D. of six experiments are represented. Statistical analysis was performed by Kruskall-Wallis test, followed by Games Howell pos-test. a vs b (p<0.05), a vs c (p< 0.01), a vs d (p<0.001).

To further characterize the potential effects of PAbs we selected H292 lung adenocarcinoma cell line, which expresses intermediate levels of both receptors, and depends on EGF to growth (data not shown). Considering that PAbs prevented ligand-dependent receptors activation, as was showed above, we specifically studied the phosphorylation state of key protein associated with HER1 and HER2 downstream signaling such as: STAT3 (p-STAT3), AKT (p-AKT) and ERK1/2 (p-ERK1/2) [3]. The treatment of H292 cells with the immune sera during 8 hours, followed by EGF stimulation, inhibited the activation of the evaluated proteins (Figure 3A). In particular, STAT3 phosphorylation was completely abrogated. In fact, PAbs diminished HER1 and HER2 expression, evaluated by flow cytometry after 24 hours of incubation (Supplementary Figure 4), suggesting that these antibodies could induce the endocytosis of these receptors. To determine if PAbs were also able to promote receptors degradation, we detected their protein levels in whole cell-lysates from H292 treated at different time points, by Western blot. As shown in Figure 3B, incubation with PAbs induced the progressive degradation of both proteins, which was complete after 24 hours of treatment. Interestingly, HER2 was drastically degraded after one hour of incubation. This could be due to its expression levels in H292 cells, which are lower than HER1 in almost one order of magnitude.

Afterward, we tested whether inhibition of receptors signaling by PAbs could impact on H292 cells viability at different times. In these experiments sera were processed to inactivate the complement to exclude its contribution to the cytotoxic effect of the sera. As shown in Figure 3C, a significant decrease in viability was detected after 48h of incubation with PAbs, this reduction became more significant with the augment of the incubation time.

### Induced HER1 and HER2 diminishment is reversed after treatment removal

**Figure 4:**
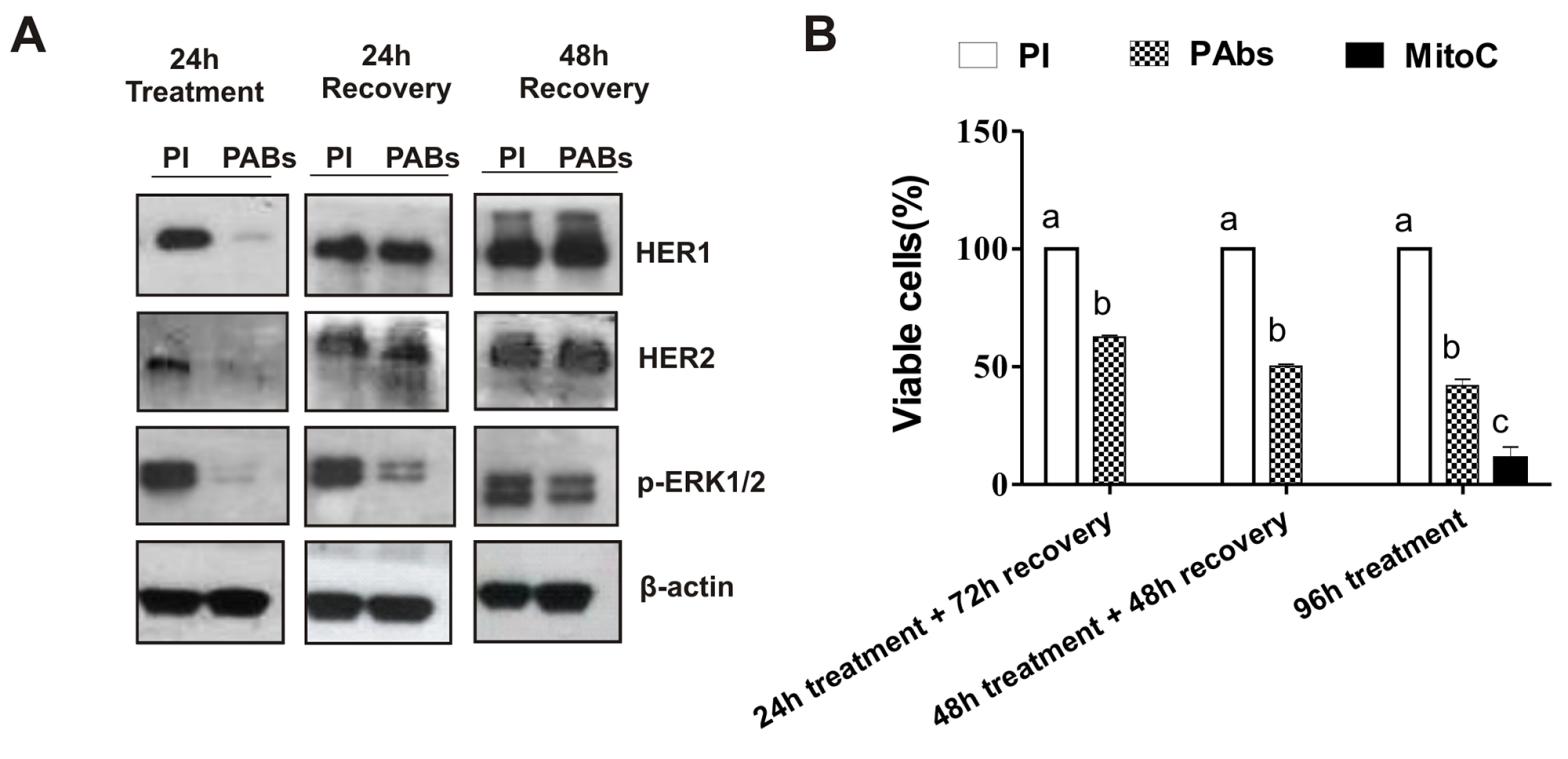
Impact of treatment removal on HER1/HER2 expression and H292 cells viability **(A)** After 24 hours of incubation with immune sera (PAbs) from day 56 (dilution 1: 100), treatment was withdrawn and cells were maintained under culture conditions for additional 24 or 48 hours. Then, cells were lysed and HER1/HER2 expression, as well as ERK1/2 phosphorylation was evaluated by Western blot. Pooled pre-immune sera (PI) were considered as negative control. **(B)** After 24 or 48 hours of incubation with PAbs (pattern box) diluted 1: 20, cells were maintained in culture conditions until complete 96 hours. PI treated cells (white box) were considered as reference of maximum viability. Cells incubated with PAbs during 96 hours were included as control of maximum viability reduction. Mean ± S.D. of six experiments are represented. Statistic was performed by a Kruskall-Wallis test, followed by Games Howell pos-test. a vs b (p<0.05)/ a vs c (p< 0.001).

The ability of H292 cells to reestablish HER1 and HER2 protein expression once PAbs were removed from culture supernatant was also determined. Cells were incubated for 24h with the immune sera to induce complete degradation of the receptors, whose protein levels were determined after removing the treatments and adding fresh culture medium to the cells. Twenty-four hours after PAbs removal, cells recovered the expression of HER1 and HER2 (Figure 4A). Furthermore, these receptors seem to be functional, since a progressive recovery of the activated ERK1/2 proteins was appreciated, which was complete after 48 hours of incubation in culture conditions.

Additionally, we measured whether the recovery of receptors expression abrogated the effect on viability induced by the incubation with PAbs. Cells were incubated with immune sera for 24 or 48 hours, after which treatments were removed and cells were maintained with fresh culture medium until they completed 96 hours of incubation. Figure 4B shows that, despite the recovered expression HER1 and HER2 in H292 cells, 24 hours of inhibition of these receptors by PAbs was enough to significantly reduce cell viability, when it was measured 72 hours later. In fact, the significance of the viability reduction did not change with the increase of the incubation time.

### Anti HER1-HER2 PAbs induce cell cycle arrest and apoptosis in H292 tumor cells

**Figure 5:**
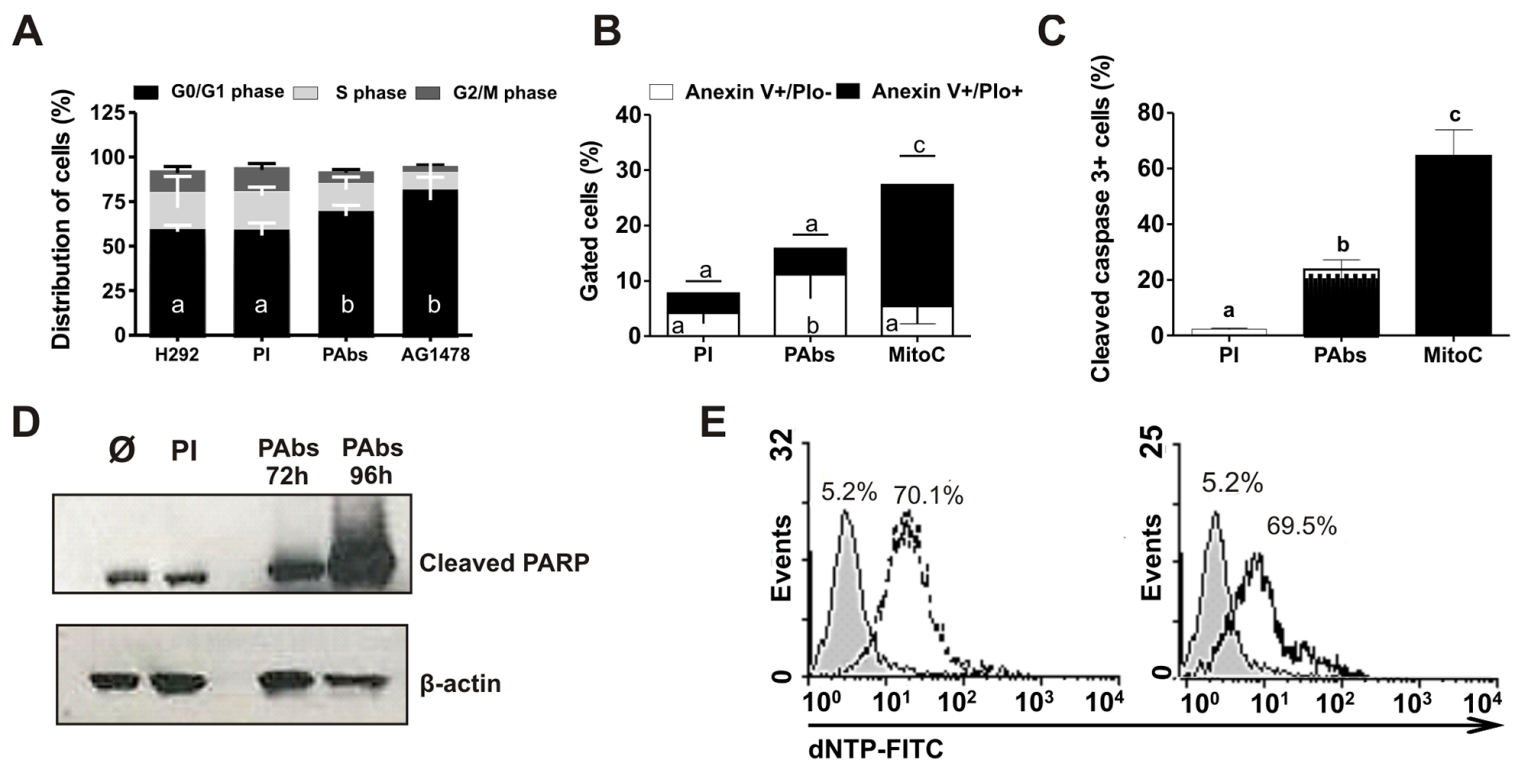
Cell cycle arrest and apoptosis induction **(A)** H292 cells were incubated during 48 hours with pooled immune sera from day 56 (PAbs) or pre-immune sera (PI) diluted 1: 20 and heated at 56°C for 30 minutes to inactivate the complement. AG1478 (1μM) was used as positive control. Cells were treated with RNAse (100μg/mL) and stained with propidium iodide (PIo) 100μg/mL. A methanalysis of three performed experiments is shown. **(B-E)** Molecular markers of apoptosis induction were measured in H292 cells treated with PAbs, diluted 1:10 and pre-incubated at 56°C for 30 minutes to inactivate the complement. PI and mitomycin C, at 10μg/mL (MitoC) were used as negative and positive controls, respectively. **(B)** Phosphatidylserine exposure (PS) was determined after 48h of treatment by Anexin V/FITC and PIo double staining (Mean ± S.D. n=5). **(C)** Caspase 3 activation was detected by flow cytometry after 72h of treatment (Mean ± S.D. n=4). **(D)** Detection of PARP cleavage by Western blot was performed after 72h or 96h of incubation. **(E)** DNA fragmentation was evaluated by TUNEL assay after 120h of incubation with PAbs (dot line histogram), PI (gray filled histogram) or MitoC (solid line histogram). Statistical analysis was performed by a Kruskall-Wallis test, followed by Games Howell post-test. a vs b p<0.05/ a vs c p< 0.01.

To determine if the reduction of H292 tumor cells viability treated with PAbs was associated with the inhibition of its proliferation, cell cycle distribution of treated cells was evaluated after 48 hours of incubation. A 10% increase in cells arrested in G0/G1 phase was detected, in comparison with the group treated with pre-immune sera (Figure 5A). The magnitude of this effect was similar to the one achieved with TKI AG1478, used as positive control of cell cycle arrest induction.

Additionally, a decrease in cell viability could also be explained by the induction of cell death. Since the cytotoxic effect demonstrated for HER1 targeting therapies is frequently connected to the induction of apoptosis [11] we measured some of the molecular markers of cell death program. Mitomycin C was included in these experiments as positive control for apoptosis induction. As shown in Figure 5B, the percentage of cells that exposed phosphatidylserine (PS) in the outer layer of their cell membranes increased after incubation with PAbs(AnexinV+/PIo- population), suggesting the promotion of the cells to early stages of death. Also, we detected an increase of 20% of cells expressing activated Caspase 3 after 72 hours of treatment (Figure 5C) which was confirmed by the progressive increase in PARP cleavage (Figure 5D). Finally, after 120 hours of treatment, we detected an increase in the number of cells with fragmented DNA (approximately 60%), in comparison with pre-immune sera and similar to cells treated with Mitomycin C (Figure 5E). These results together indicate that generated PAbs induce apoptosis in H292 cells, so this mechanism could also contribute to the observed reduction in cell viability.

**Figure 6:**
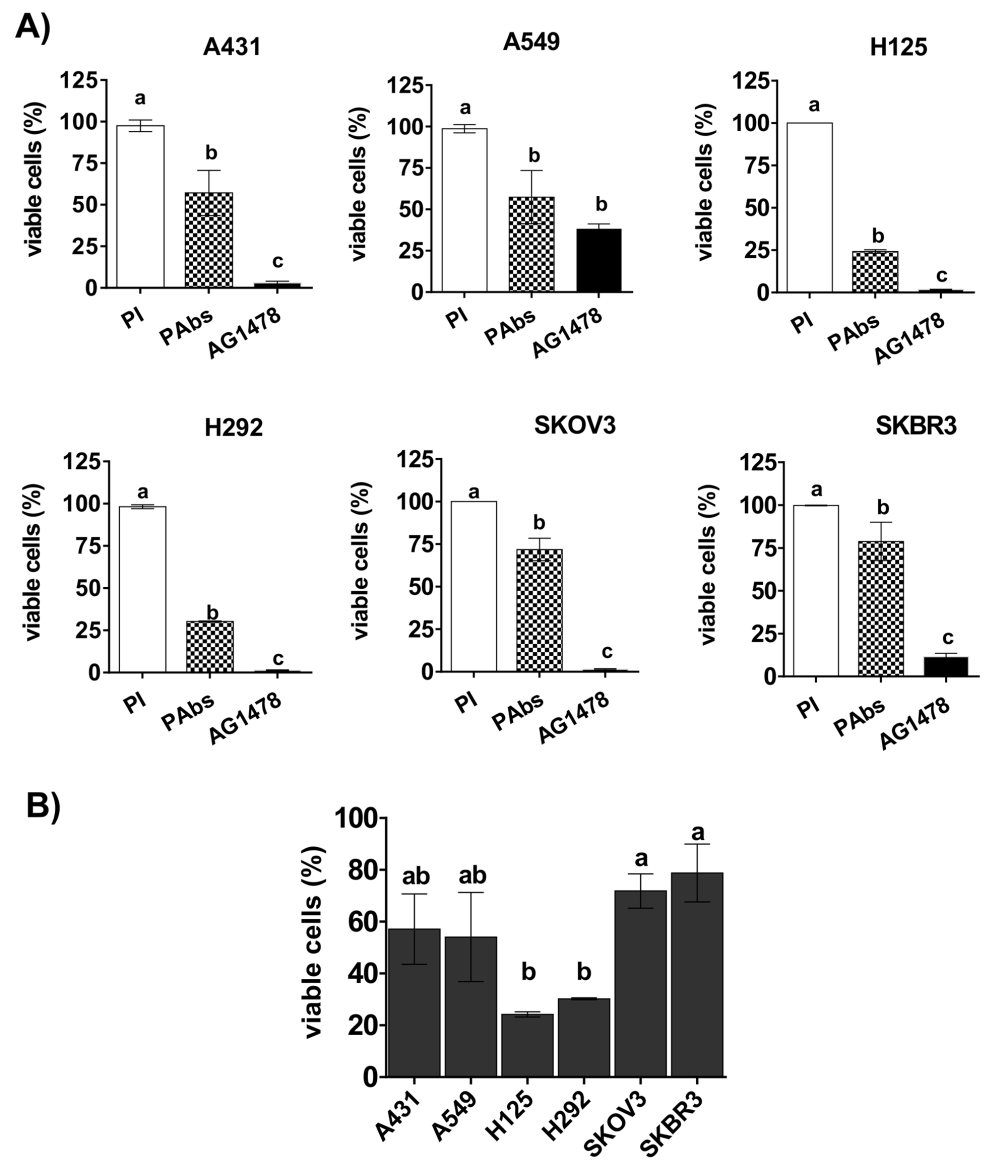
Reduction of cell viability in a panel of tumor lines **(A)** A431, A549, H125, H292, SKOV3 and SKBR3 tumor cells were treated for 96h with immune sera from day 56 (PAbs, pattern box) diluted 1: 20 and pre-incubated at 56°C for 30 minutes to inactivate the complement. Cells treated with pooled pre-immune sera (PI, white box) were considered as reference of maximum cell viability, while AG1478 (10μM) was included as positive control of cell viability reduction (black box). Mean ± S.D. of four experiments are represented. **(B)** The graphic represents the comparison of the viability decrease among the evaluated cell lines: Mean ± S.D. of the percentage of viable cells (with regard to PI treated cells) corresponding to four experiments is represented. Statistical analyses were done using Kruskall-Wallis test, followed by Games Howell pos-test. a vs b (p<0.05), a vs c (p< 0.01).

To examine a possible relation between the expression levels of HER1 and HER2 and the impact of PAbs on cell viability, a total of five tumor lines with differential expression of both receptors were incubated with PAbs during 96 hours and viability was tested as described. As shown in Figure 6, PAbs significantly decreased cell viability in all studied cell lines, including A549 cells which are characterized by a constitutively activating mutation of the ERK1/2 cascade, the KRas isoform, which has been associated with resistance to MAbs [20]. Remarkably, the most significant effects were obtained with H125 and H292 cells, which possess the lowest expression levels of both receptors among examined lines.

## DISCUSSION

HER1 and HER2 overexpression has being reported in many types of cancer and correlates with poor prognosis of the patients, supporting the role of these receptors in tumor biology. Passive therapies targeting HER1 and HER2 oncogenes have been validated in clinical trials impacting on patients survival [2]. However, almost inevitably tumor resistance emerges [21] supporting the search of new approaches capable of deal with the complexity of tumor signaling network. Since heterodimers of these receptors are more potent signaling-transducing complexes than homodimers [22], and HER2 is always conformational competent for interaction (which makes it the favorite partner for dimerization) we decided to target simultaneously HER1 and HER2, through endogenous PAbs. Indeed, using PAbs instead of MAbs, is the best way to harness the immune system response in order to simultaneously target multiple epitopes of several antigens.

We evaluated this approach by immunizing mice with ECD of the human variants of HER1 and HER2, with 87% and 85% of sequence identity to their murine variants, respectively. Induced PAbs recognized full length receptors expressed on cell surface despite the fact that immunization was performed with truncated receptors, as was demonstrated for HER1 specific PAbs in our previous studies [23]. Their capacity to diminish the viability of H292 lung carcinoma cells was associated with the inhibition of downstream cascades: ERK1/2 (MAPK), PI3K/Akt and Jak/STAT. It has been widely described that the exacerbated activation of these cascades produces an increase in the proliferation and survival of tumor cells [1]. Interestingly, after 8 hours of incubation, this inhibition was more pronounced for STAT3 and Akt proteins, while ERK1/2 phosphorylation was only partially affected. Nevertheless, sustained blockade of HER1 and HER2 for longer incubation times (24 hours) eventually leads to a complete inhibition of ERK1/2 phosphorylation, as shown in Figure 5A. In the case of STAT3, further studies should be done to determine the implication of its inhibition in the intrinsic inflammatory program that is often triggered by tumors to sustain proliferation and survival [7].

The impairment of these cascades could be associated not only with the blockade of ligand-dependent activation of HER1 through homo and heterodimers formation, but also with the degradation of both receptors promoted by PAbs. Despite HER2 is considered more resistant to degradation than HER1, and protects its partners when heterodimers are formed [24], after 1 hour of incubation of H292 cells with PAbs, HER2 expression was almost undetectable by Western blot. Previous studies have associated the antitumor effect of the combination of anti-HER1 [25] or anti-HER2 [14] with receptor internalization and consequent degradation in lysosomes. For instance, Maron *et al.* described that the combination of MAbs against HER1 an HER2 promotes degradation of these receptors. However longer incubation times were considered in this study [16], reinforcing the idea that PAbs may have a stronger impact on receptors intracellular trafficking and recycling. However, we also observed that HER1 and HER2 expression and signaling was completely reestablished after 48 hours of treatment withdraw, suggesting the relevance of a constant presence of antibodies in the tumor vicinity. This effect could be important in a clinical setting, and suggest and advantage for endogenous PAbs with regard to passive immunotherapies (MAbs), which are eliminated from the organism in a few days, while memory clones of antibodies-secreting B cells induced by vaccination could determine a prolonged production of these antibodies. Beyond reestablishment of receptors expression, is was also observed that twenty-four hours of treatment were enough to impact the tumor cells viability measured at long term (Figures 3D and 5B). Nevertheless, the restoration of HER1 and HER2 might eventually determine an increase in H292 cells viability after treatment removal, which should be evaluated in future studies.

The reduction in H292 cells viability induced by PAbs was associated with a blockade of cell cycle progression, as well as with the induction of cell death with apoptotic features. HER1 and HER2 signaling is clearly linked to sustained proliferation of tumor cells, since they up-regulate the expression of Cyclin D1 during most phases of cell cycle [26]. However the induction of apoptosis-related molecular events observed in H292 cells is consistent with the lack of survival stimuli through PI3K/Akt pathway, which could induce the activation of an apoptotic program [27].

Additionally, PAbs had a significant negative impact on the viability of tumor lines with different expression levels of HER1 and HER2. The study included cells which overexpress HER1 or HER2, others which don´t overexpress any of the receptors, and also tumor cells with constitutive KRAS mutation. The fact that PAbs inhibited cell viability in all these scenarios suggests its potential effect on epithelial tumors *in vivo*, since these malignancies are characterized by regional heterogeneity in the expression and activation patterns of these molecules [28, 29]. The highest viability reduction provoked by PAbs, was evidenced in the cells lines with lower levels of both receptors: H292 and H125, which could be explained by several facts.

Our first hypothesis is sustained in previous evidences suggesting that the frequency of receptors heterodimers in tumor lines is lower for those expressing higher levels HER1 or HER2, since the equilibrium is displaced toward homodimerization of the overexpressed partner [30]. The inhibition of HER2 phosphorylation by PAbs, when cells are stimulated with a HER1 ligand (EGF) suggests its capacity to disturb the formation of HER1-HER2 heterodimers in H292 cells. Then, it could be possible that cells with low expression levels have higher dependence on heterodimers signaling rather than one receptor activation and, therefore, they could be more sensitive to PAbs. However, our results are not conclusive in this sense, and further studies should be conducted to stablish the effect of PAbs on the HER1-HER2 heterodimerization frequency in a panel of treated tumor lines with differential expression of both receptors.

Another explanation could be related with the complete degradation of HER1 and HER2 observed in intermediate-expressing H292 cells after few hours of incubation. The magnitude and kinetic of this effect might be dependent on the expression levels of the receptors. Also, the less responsiveness to some tumor lines might be due to an aberrant expression of molecules typically associated with resistance to anti-HER1/2 targeting therapies such as c-Met, which is expressed in A431 tumor line [31], IGF-1R, expressed in SKBR3 tumor line [32], or another HER family members like HER3, overexpressed in SKBR3 tumor lines [33], as well as mutations driving constitutive activation of downsignalling cascades like KRas isoform detected, in A549 line [34].

The impairment of cell viability in tumor lines with intermediate levels of HER1 and HER2, as well as lines with resistance driving mutations suggest a set of patients that could benefit from this therapeutic approach, for which the clinical benefit of approved MAbs has been limited. In this sense, it has been reported for MAbs with intermediate affinity like nimotuzumab, that overexpression of HER1 is critical to elicit a significant anti-tumoral effect both *in vitro* and *in vivo*, since bivalent binding of the antibody is required [10]. In a similar way, trastuzumab, an anti-HER2 specific MAb approved for clinical use, has a positive correlation between the response to treatment and the HER2 expression levels in patients with metastatic breast cancer [35]. The presence of constitutive activating mutations like EGFR-T790M, BRaf and KRas drives resistance to therapies targeting HER1 or HER2 [36, 37].

In summary, simultaneous targeting multiple epitopes of more than one HER family member can effectively impair tumor cells proliferation and survival, particularly in cells which don´t overexpress these receptors. This fact advises a differentiation strategy whit regard to clinically approved MAbs. Of note, similar titers of PAbs can be generated in F3II tumor-bearing as well as in healthy mice, and they equally recognize the antigens expressed on tumor cells. Selecting the right adjuvant in cancer has a major role considering the immunosuppressive environment triggered by tumors or anti-cancer therapies. The adjuvant used in the formulations was VSSP (very small-sized proteoliposome) derived from the outer membrane of *Neisseria Meningitidis* and it has proved to act as immune system-modulator by reducing the regulatory function of myeloid-derived suppressor cells [38]; and therefore diminish the suppression caused by tumor microenvironment. This is relevant considering that in clinical scenario, patients are mostly severely immunosuppressed by tumor burden or therapies [39].

Despite the high sequence identity between human and murine HER1 and HER2, these variants are not completely homologous. Further studies should be addressed including autologous bivalent vaccine to evaluate the potential toxicity of the induced immune response, since the combination of cetuximab and trastuzumab has evidenced increased toxicity in the clinical setting [40]. However, our previous studies showed the proof of principle of autologous vaccination, immunizing with murine EGFR adjuvated in VSSP. This vaccine circumvented the tolerance to this self-protein, inducing humoral and cellular immune response, with antimetastatic effect *in vivo* [41] without evidences of toxicity [42, 43]. Additionally, break of tolerance against the human variant of HER1 using VSSP as adjuvant, has been also evidenced in closest models like monkeys, without any sign of toxicity [44]. Furthermore, lack of toxicity has been detected in thousands of patients vaccinated with different tumor associated antigens [45].

Taken together, these evidences suggest the potential efficacy of this multi-target PAb’s approach for the design of a new cancer vaccine.

## MATERIALS AND METHODS

### Antibodies and reagents

Antibodies specific for p-EGFR (Tyr1068), p-ERK1/2 (Thr202, Tyr 204), p-Akt (Ser473), p-STAT3 (Tyr705) and antibodies to total EGFR, ERK1/2, Akt, STAT3 and PARP used for Western Blot experiments were purchased from Cell Signaling Technology Inc. (USA). Anti p-HER2 (Tyr1248) and total HER2 were obtained from Santa Cruz Biotechnology (USA). An antibody specific for cleaved Caspase 3 conjugated to Alexa-Fluor®488 was used for flow cytometry experiments (Cell Signaling Technology Inc.). The recombinant human EGF was obtained from the Genetic Engineering and Biotechnology Institute (Cuba). Anti-HER1 MAb nimotuzumab (TheraCIM hR3) was manufactured at the Center of Molecular Immunology (Cuba) while trastuzumab (Herceptin) was purchased from Roche (Germany). Mitomycine C and AG1478 were acquired from Sigma (USA).

### Cell lines

Human epidermoid carcinoma A431 (ATCC CRL-1555), human lung adenocarcinomas A549 (ATCC CRL-1555) and H292 (ATCC CRL-1848); breast carcinoma MDA-MB468 (ATCC HTB-132), PC3 prostate carcinoma (ATCC CRL-1435), SKOV3 ovarian carcinoma (ATCC HTB-77) and Raji lymphoma (ATCC CCL-86) cell lines; were grown in DMEM-F12 (Gibco, USA) supplemented with 10% fetal calf serum (FCS), (Hyclone, USA). SKBR3 breast carcinoma (ATCC HTB-30) was grown in RPMI (Gibco, USA) at 10% FCS. The lung adenocarcinoma H125 and the breast adenocarcinoma MCF, transfected to ectopically express HER2, were gently donated by the Molecular Biology Department of MPI (Germany). HEK 293 transfectants producing extracellular domains (ECD) of HER1 or HER2, were adapted to growth in CPCHO medium supplemented with insulin (5μg/mL) [46].

### Antigens

The ECD of human variants of the receptors were used in the immunization protocols. HER1 extracellular domains (HER1-ECD) was obtained as previously detailed [41]. HER2-ECD (a.a 1-653) was purified from supernatants of HEK293 transfectants using immobilized trastuzumab.

### Immunization protocols

Female and BALB/c mice, aged 8–12 weeks old, were purchased from the National Center for Laboratory Animals Production (CENPALAB, Havana, Cuba). All mice were kept under pathogen-free conditions. Animal experiments were approved by the Center of Molecular Immunology’s Institutional Animal Care and Use Committee (CIM, Havana, Cuba).

### Generation of specific polyclonal antibodies against HER1 and HER2

BALB/c mice (n=5) were immunized four times biweekly with 100μg of (HER1-ECD) or HER2-ECD (monovalent vaccines). Another two groups received a mixture of 100μg or 400μg of both ECDs (bivalent vaccines). All the preparations were adjuvated in very small size proteoliposome (VSSP, 200μg per mouse) derived from the outer membrane of *Neisseria meningitides*, containing N-acetyl GM3 ganglioside [47]. Sera was obtained from blood extractions performed on days -2 (pre-immune serum), 35, 56, 72 and 102.

### PAbs generated in F3II-bearing mice

BALB/c mice (n=3) were inoculated with F3II tumor cells (10^6^/mouse) on day 0. After tumors were detectable, mice were immunized twice (at days 10 and 25 of the schedule) with 400μg of HER1 and HER2 adjuvated in 200μg of VSSP. A second group of tumor-free BALB/c mice (n=3) were immunized as described (400μg of HER1 and HER2 were employed) and considered as a control. At day 30 sera were obtained from blood and animals were sacrificed considering tumor burden.

### ELISA

Microtiter plates (High binding, Costar, USA) were coated with 5μg/mL of EGFR-ECD or HER2-ECD in carbonate buffer, 0.1M, pH 9.6, and incubated overnight at 4°C. Plates were blocked with 5% FCS in PBS/0.05% Tween 20. Sera dilutions (immune or pre-immune) were incubated for 1 h at 37°C for PAbs titration. Alkaline phosphatase-conjugated goat anti mouse IgG antibody (Sigma, USA) was added and incubated for 1h at 37°C. After addition of p-nitrophenylphosphate (1 mg/mL) (Sigma, USA) in diethanolamine buffer pH 9.8, the absorbance at 405nm was measured using a microwell system reader (Organon Teknica, Salzburg, Austria). ELISA test background was two times the absorbance at 405nm of pre-immune sera, used as negative control.

### Flow cytometric analysis

Cells were stained with specific antibodies during 20 min. Anti-EGFR PAb were diluted 1:200-1:3200. MAbs nimotuzumab or trastuzumab were used at 10μg/mL and 1μg/mL, respectively. Staining was followed by incubation with FITC-linked goat anti-mouse IgG or FITC-linked goat anti-human IgG for murine PAbs and human MAbs, respectively (Sigma). All analyses were performed using a Gallios flow cytometer (Beckton Dickinson, San Jose, CA, USA) and data were processed using Kaluza 1.2 software apart from Cell cycle analyses in which FlowJo 5.6 software was used.

### Western blot

H292 cells were grown to 70% of confluence in 12-well plates (Greiner) and incubated with serum-free culture medium for 12h. The medium was changed to fresh serum-free medium containing immune or pre-immune sera diluted 1:100. Tyrosine kinase inhibitor AG1478 was used as EGFR inhibition control (10μM). Cells were incubated with human EGF (100 ng/ml) for 10min, lysated and then transferred to polyvinylidine difluoride membranes (GE Healthcare, USA) for immunoblotting.

### Immunoprecipitation

Protein A beads were co-incubated overnight at 4°C with A431 lysates and pre-immune or immune sera. As positive controls for HER1 and HER2 precipitation respectively, 2μg of nimotuzumab and trastuzumab were used instead serum. The precipitated was extensively washed, applied to 7.5% SDS-PAGE, transferred to PVDF membranes and immunobloting was performed to detect HER2 and HER1 with specific antibodies as described.

### Viability assays

Flat bottomed 96-well microculture plates were seeded with H292, H125, A549, SKOV3, SKBR3 (10^4^) or A431 (5 × 10^3^) cells in 100μL/well and grown in DMEM-F12 (RPMI for SKBR3) supplemented with 10% FCS. Twenty-four hours later culture medium was removed and cells were incubated DMEM-F12 (RPMI for SKBR3) supplemented with 1% FCS in the presence of sera diluted 1:20. Previous to addition, sera were incubated at 56°C for 30 minutes to inactivate the complement. After 96h of incubation at 5% of CO_2_, cell viability was measured by the modified colorimetric MTT (3-[4,5-dimethylthiazol-2-yl]-2,5diphenyl tetrazodium bromide) assay. Half of the media was replaced by 50μL/well of MTT (2mg/mL) and plates were incubated under culture conditions for 4h. Formazan crystals were dissolved by the addition of 100μL/well of dimethyl sulphoxide, followed by 15 of incubation at room temperature. Absorbance was measured at 540 nm and the reference wavelength (620 nm) absorbance subtracted. Background control contained only culture medium without cells. Cells without treatment, as well as cell treated with pre-immune sera were included as maximum cell growth control. In these experiments, Mitomycin C was used as a cytotoxicity control.

### Cell cycle analysis

H292 cells were plated (2.5×10^5^) in DMEM 10% FBS in 6-well plates. Twelve hours later cells were incubated for additional 48 h with immune sera diluted 1:10 or AG1478 (10μM) used as positive control. The sera were processed to inactivate the complement by incubation at 56 °C for 30 minutes. Next, cells were fixed with ice-cold methanol/acetone (4:1) and stained with a solution containing 100μg/mL of propidium iodide (PIo, Sigma) and 50μg/mL of RNAse (Sigma).

### Measurement of molecular determinants for apoptosis

H292 cells were plated (1×10^5^cell/well) in DMEM 10% FBS in 12-well plates. Twelve hours later, cells were treated with pre-immune or immune sera diluted 1:10 (and processed to inactivate the complement by incubation at 56°C for 30 minutes) or Mitomycine C (10μg/mL). To determine phosphatidylserine exposure cells were incubated for 48 h and then stained with an Anexin V-Propidium iodide kit, according to manufacturer’s instructions. Capase 3 cleavage was measured after 72h of incubation, following a fixation step using Cytofix/Cytoperm solution included in the Golgi Stop set of reagents. To analyze PARP activation, cells were treated as described by 72 or 96h, homogenized, and levels of cleaved PARP were determined by Western blot. DNA fragmentation was measured after 120h of treatment. In situ, cell death detection reagents were used according to manufacturer’s instructions (TUNEL, Roche) and analyzed by flow cytometry.

### Graphical representation and statistical analysis

GraphPad Prism program (version 7.0) was used for graphical representation of the results. Statistical analysis were performed using the IBM SPSS Statistic 19 program. All data were analyzed using non-parametric tests, since we didn’t obtain normality of the values or variance homogeneity by Kolmogorov-Smirnov and Levene tests, respectively. For PAbs titration the Mann Whitney-U non parametric test was selected. Kruskall-Wallis test and Games-Howell post-test were used to determine statistical differences among media from the treatment and control groups in viability determination assays, cell cycle distribution and evaluation of molecular markers of apoptosis. In graphic representations, significant differences were highlighted assigning the letter “a” to indicate the greater media among analysed groups. Significantly different samples were emphasized with letters “b” (p<0.05), “c” (p<0.01) or “d” (p<0.001). When the differences were non-significant, samples were identified using the same letter.

## SUPPLEMENTARY MATERIALS FIGURES



## Abbreviations

HER1 and HER2: epidermal growth factor receptor family members
ECD: extracellular domain
Abs: antibodies
PAbs: polyclonal antibodies
MAbs: monoclonal antibodies
BV: bivalent vaccine
MV: monovalent vaccine
PI: pre-immune sera
PIo: propidium iodide
TKIs: tyrosine kinase inhibitor
MAPK: mitogen activated protein kinase
PI3K: phosphoinositol kinase 3
STAT3: signal transducer and activator of transcription 3
PS: phosphatidylserine
VSSP: very small-sized protoeliposomes
MFI: mean fluorescence intensity
SD: standard deviation
