# Supplementary material for: Anti-proliferative and pro-apoptotic effects induced by simultaneous inactivation of HER1 and HER2 through endogenous polyclonal antibodies
Source: Oncotarget. 2017 Aug 3;8(47):82872–84. doi: 10.18632/oncotarget.19958 (PMC5669935; doi:10.18632/oncotarget.19958)
Supplement: Supplementary file 1 [file oncotarget-08-82872-s001.pdf]

Anti-proliferative and pro-apoptotic effects induced by simultaneous inactivation of HER1 and HER2 through endogenous polyclonal antibodies

SUPPLEMENTARY MATERIALS

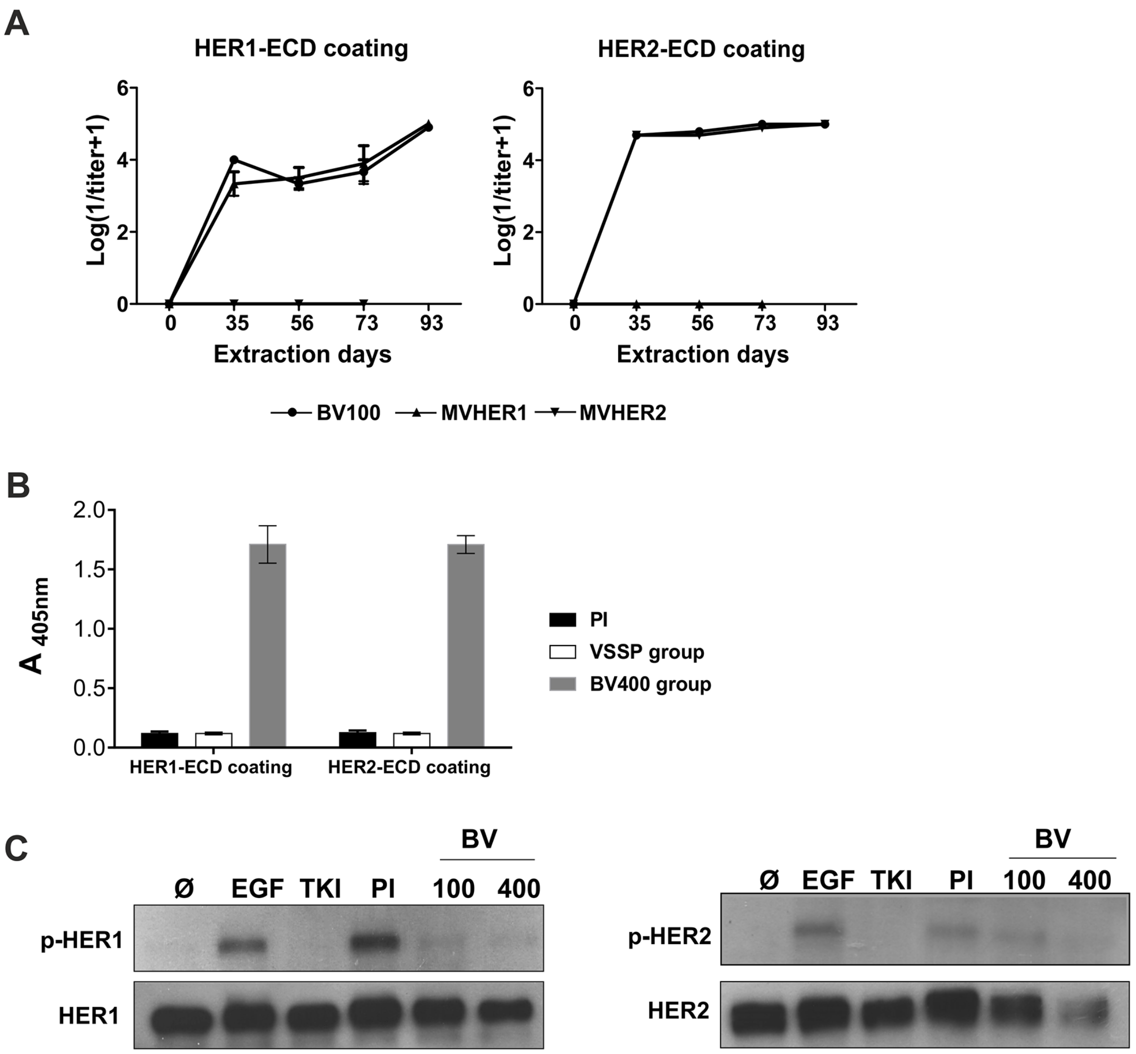

Supplementary Figure 1: Comparison of the specific antibodies responses induced by monovalent or bivalent vaccines. **(A)** BALB/c mice (n=5) were immunized four times biweekly (sc.) with 100µg of HER1-ECD (MVHER1), HER2-ECD (MVHER2), or a mixture of both ECDs (BV100) adjuvated with 200µg of VSSP. Sera were obtained from blood collected at days -2, 35, 56, 73 and 93. Titration of HER1 and HER2 specific IgG antibodies in sera was performed by ELISA. Data was Log transformed (1/titer + 1) for graphic representation. **(B)** BALB/c mice (n=4) were immunized four times biweekly (sc.) with 200µg of VSSP, used as negative control of specific antibodies induction (VSSP group). Sera were obtained from blood collected at days -2 and 35. The presence of anti-HER1 or HER2 IgG antibodies was determined by ELISA, and compared with time-equivalent immune sera obtained from mice immunized with the bivalent vaccine, employing 400µg of both ECDs (BV400 group). A pool of pre-immune sera was included as negative control (PI). The figure represents the Mean ± S.D. corresponding to four individual values. **(C)** H292 cells were incubated during one hour with pooled immune sera from mice immunized with BV100 or BV400 (diluted 1:100) and further stimulated with EGF (10 min, 100ng/mL). HER1 and HER2 expression and phosphorylation status was evaluated by Western blot. PI was used as negative control in this assay.

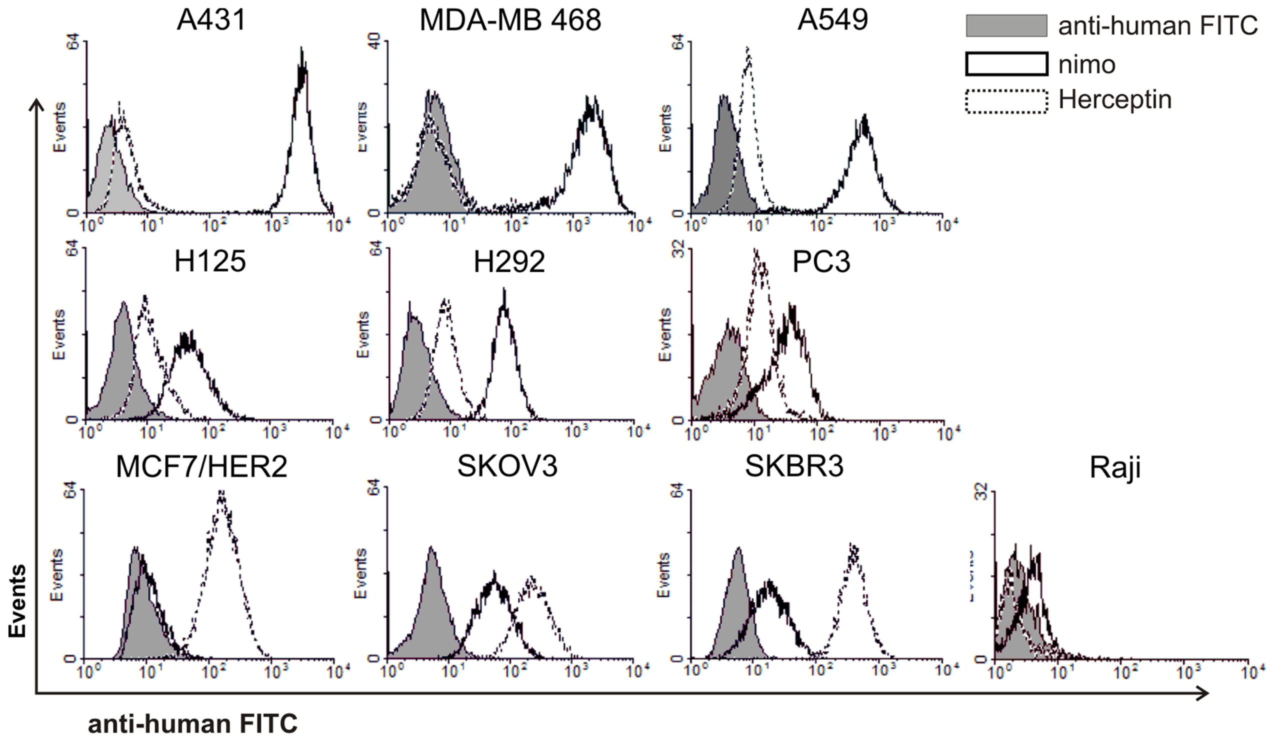

Supplementary Figure 2: Confirmation of the expression levels of HER1 and HER2 in a panel of human tumor lines. Cells lines (n= 10) were incubated with nimotuzumab (nimo) at 10µg/mL (continuous line histograms) or trastuzumab (Herceptin) at 1µg/mL (dotted line histograms) followed by goat anti-human IgG FITC staining. Cells stained directly with the conjugated were included as negative control (gray filled histograms).

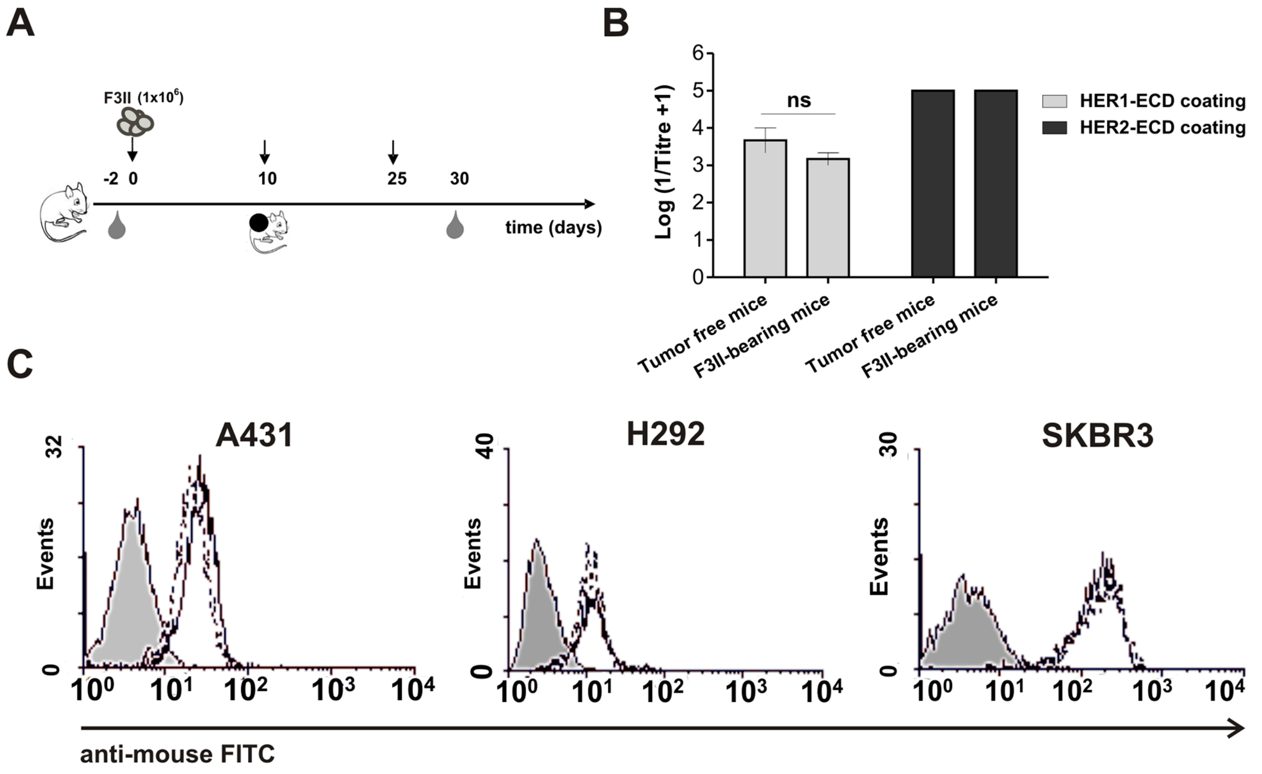

Supplementary Figure 3: Induction of specific antibodies response against HER1 and HER2 in F3II tumor-bearing mice. **(A)** Schematic representation of mice treatment. BALB/c mice (n=3) were inoculated s.c with 1x106 F3II cells. After tumor detection (day 10) mice were immunized three times biweekly with 400µg of HER1-ECD and HER2-ECD adjuvated in 200µg of VSSP. **(B)** Sera were obtained from mice euthanized at day 30. Titration of specific IgG antibodies against HER1 and HER2 was performed by ELISA. Data was Log transformed (1/titer + 1) for representation. Statistical differences among the groups were determined using the non-parametric Mann-Whitney U test. **(C)** A431, H292 and SKBR3 tumor cells were incubated with immune sera (diluted 1: 100) from healthy (black line histograms) or tumor-bearing mice (dot line histograms) and compared with pre-immune sera (gray filled histograms) included as negative control.

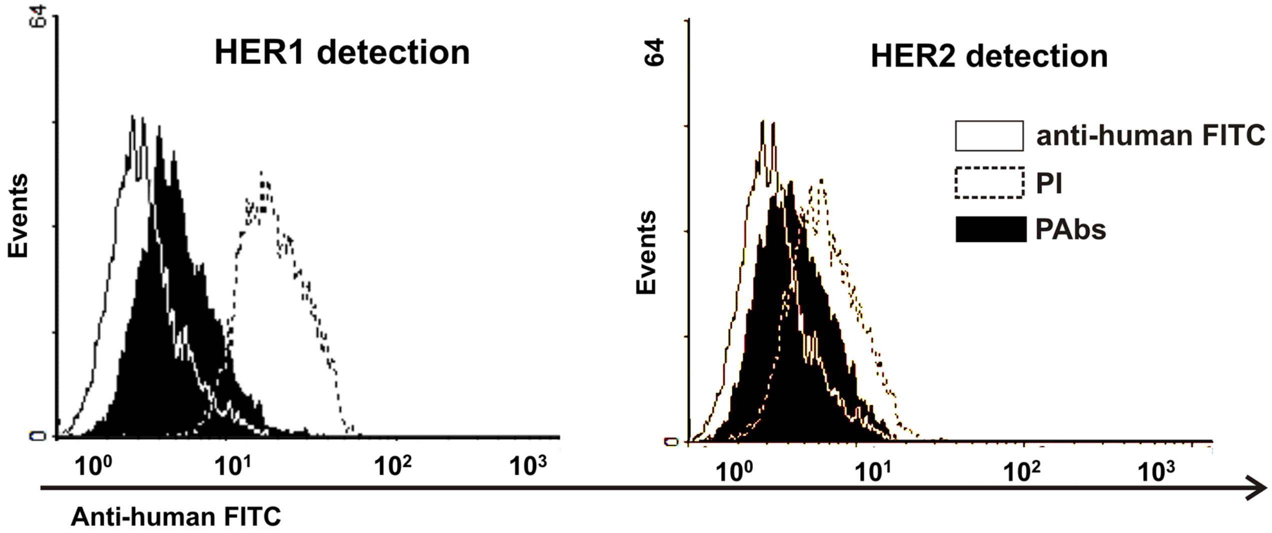

Sample	MFI (AU)
anti-human FITC	3.40
PI incubation	20.03
PAbs incubation	5.09

Sample	MFI (AU)
anti-human FITC	3.30
PI incubation	8.54
PAbs incubation	4.32

Supplementary Figure 4: Inhibition of HER1 and HER2 surface expression by PAbs. H292 cells were incubated for 24 hours with immune sera from day 35 (PAbs) or with pre-immune sera (PI), both diluted 1: 100. HER1 and HER2 surface expression was determined by flow cytometry. Staining of the cells proceeded as described in Supplementary Figure 2. Cells incubated with PI are represented by dot line histograms, while those incubated with PAbs correspond to black filled histograms. Cells stained directly with the conjugated were included as an additional control (continuous line histograms). Tables below summarize the Mean Fluorescence Intensity (MFI) corresponding to each sample.
